# Robot-assisted laparoscopic hepatectomy for liver metastasis from clitoral malignant melanoma: a case report

**DOI:** 10.1186/s40792-024-02058-7

**Published:** 2024-11-11

**Authors:** Hitoshi Iwasaki, Shinji Itoh, Norifumi Iseda, Yuriko Tsutsui, Takuma Izumi, Yuki Bekki, Shohei Yoshiya, Takamichi Ito, Takeo Toshima, Takeshi Nakahara, Tomoharu Yoshizumi

**Affiliations:** 1 Department of Surgery and Science, Graduate School of Medical Sciences, Kyushu University, Fukuoka, 812-8582 Japan; 2 Department of Dermatology, Graduate School of Medical Sciences, Kyushu University, Fukuoka, Japan

**Keywords:** Adjuvant chemotherapy, Liver metastasis, Malignant melanoma

## Abstract

**Introduction:**

Malignant melanomas occur most commonly in the skin, mucous membranes, or choroid. Clitoral malignant melanomas are extremely rare. Stage IV malignant melanomas have a poor prognosis, and molecularly targeted agents or immune checkpoint inhibitors are recommended. However, surgical resection is reportedly a valid option for improving the prognosis of patients with oligometastases, defined as a small number of metastases that can be completely resected. In this report, we describe hepatic resection for a recurrent liver metastasis in a patient who had undergone removal of a clitoral malignant melanoma 9 years previously.

**Case presentation:**

An 82 year-old woman presented with a black nodule on her clitoris. Total resection of the nodule resulted in a diagnosis of clitoral malignant melanoma (pT4bN0M0, pStage IIC; UICC 8th edition). A follow-up computed tomography scan 4 years later revealed a single 5 mm mass in the lower lobe of the right lung, prompting partial resection of the right lung. Pathological examination of the operative specimen revealed a pulmonary metastasis of malignant melanoma. The patient was treated with pembrolizumab monotherapy as adjuvant chemotherapy for 1 year. A follow-up computed tomography scan 9 years after surgical removal of the primary lesion revealed an 18 mm mass in segment II of the liver, prompting robot-assisted laparoscopic left lateral sectionectomy. The provisional diagnosis of metastatic malignant melanoma in the liver was confirmed by histopathological examination of the operative specimen. The patient was treated with pembrolizumab monotherapy as postoperative adjuvant chemotherapy for 1 year. No further recurrence was detected at the 1.5 year follow-up.

**Conclusion:**

We performed hepatectomy for oligometastasis of clitoral malignant melanoma, an extremely rare entity. Surgery has the potential to prolong the prognosis of patients with oligometastasis.

## Introduction

Malignant melanomas, which are caused by malignant transformation of melanocytes, are known to form on the skin, mucosa, and choroid. Mucosal malignant melanomas of the female genitalia account for fewer than 1% of all malignant melanomas, and clitoral malignant melanomas occur particularly infrequently. Malignant melanomas have the highest mortality rate of all skin cancers; the 5 year survival rate for Stage IV malignant melanoma is 24.6% [1]. Molecularly targeted agents or immune checkpoint inhibitors are generally recommended for unresectable Stage IV malignant melanoma. However, in patients with oligometastasis, defined as a small number of metastases that can be completely resected, surgical resection is reportedly a valid option for improving the prognosis [2–4]. In this report, we describe our experience with a patient who underwent hepatic resection for a metastasis from a clitoral malignant melanoma and received adjuvant chemotherapy thereafter.

## Case presentation

**Fig. 1:**
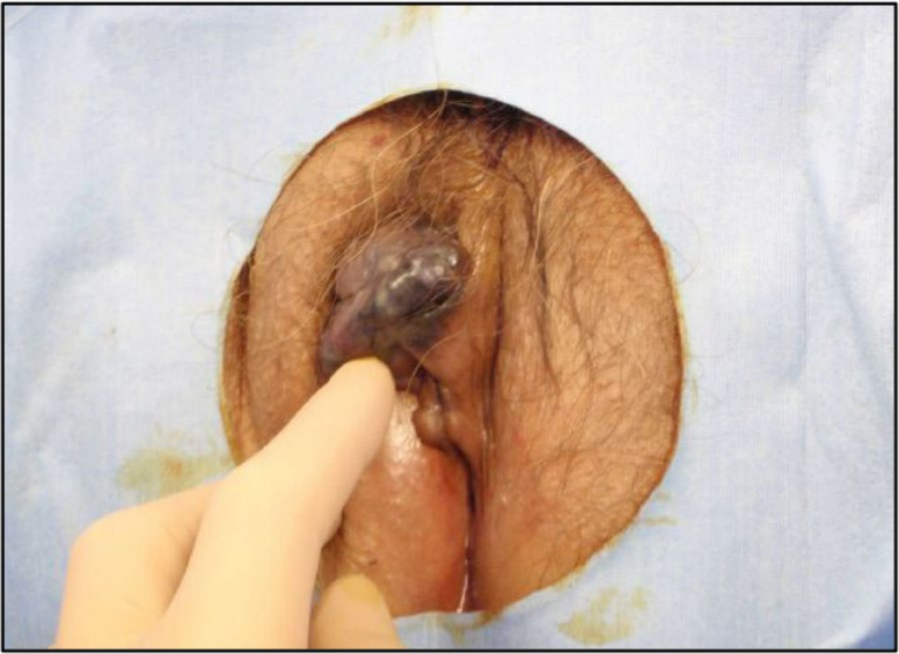
Photograph showing a black mass the size of the tip of the index finger head on the clitoris. The provisional diagnosis was malignant melanoma

**Fig. 2:**
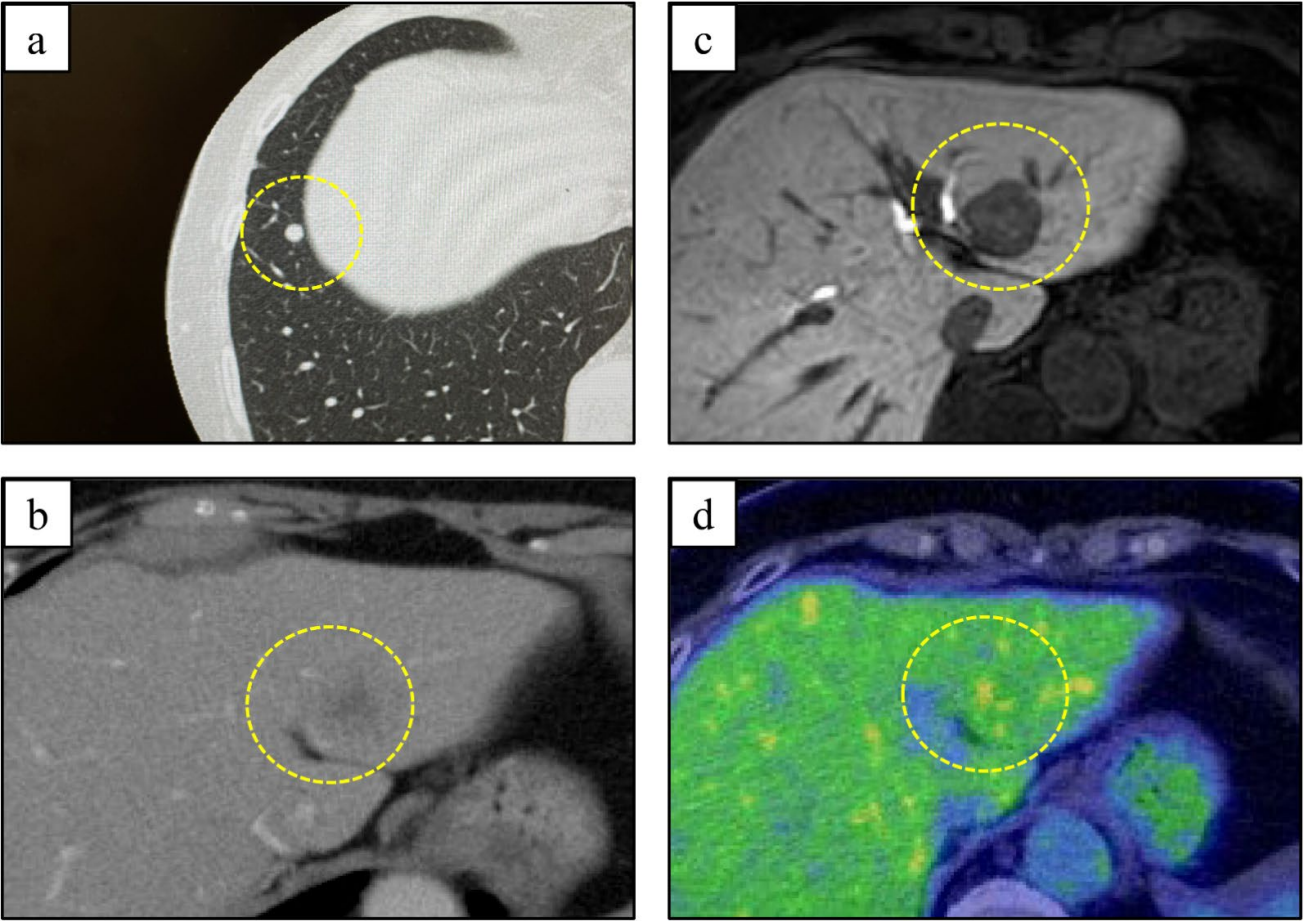
Imaging findings of the metastases. **a** Computed tomography image showing a single 5 mm mass (dashed line) in the lower lobe of the right lung. **b** Computed tomography image obtained 9 years after the first surgery showing a single 18 mm hypo-absorptive zone in liver segment II (dashed line). **c** Magnetic resonance imaging scan showed high intensities in the main lesion on T1-weighted imaging, suggesting melanin deposition (dashed line). **d** F-18-fluoro-2-deoxyglucose (FDG) positron emission tomography–computed tomography revealed mild FDG deposition in the main tumor with a maximum standardized uptake value of 3.76 (dashed line)

**Fig. 3:**
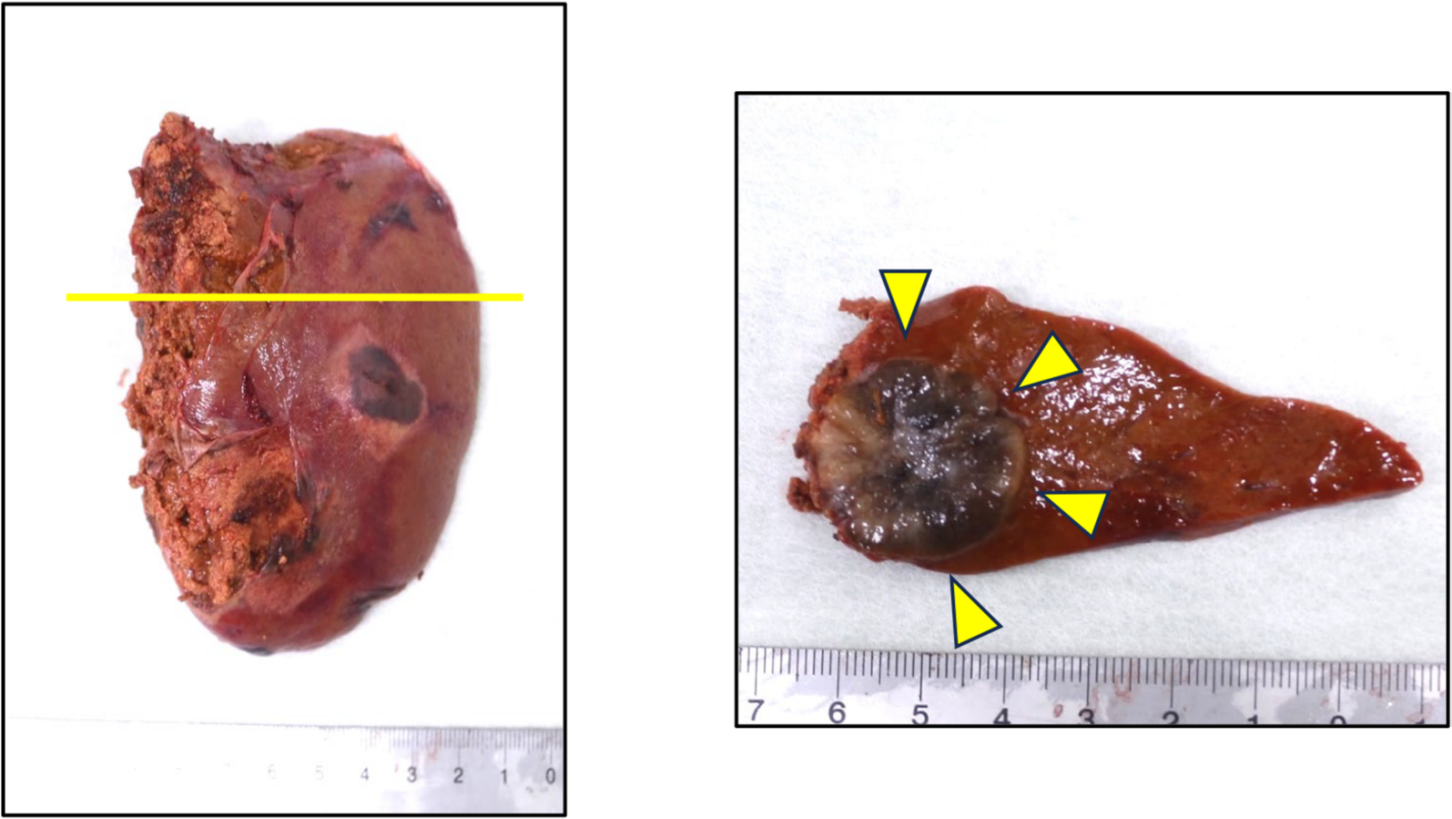
Macroscopic findings of hepatic lesion. A black 2.4 × 2.5 cm tumor is visible in segment II of the liver (arrows)

**Fig. 4:**
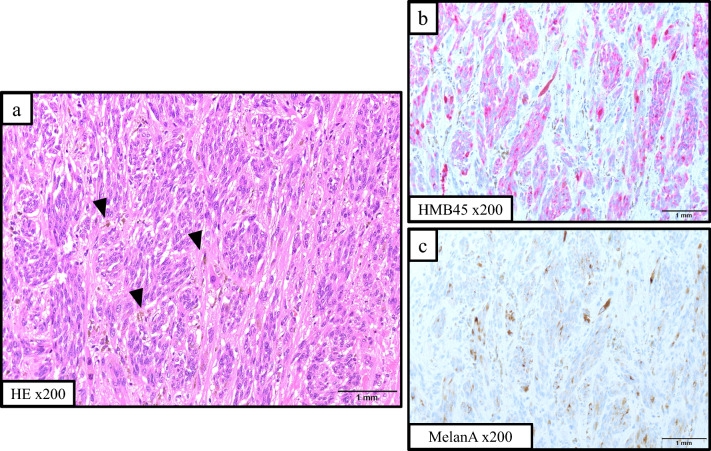
Histopathological and immunostaining findings. **a** Photomicrograph showing oval to spindle-shaped nuclei in tumor cells that are growing in sheets and nests. Some of the cells contain melanin (arrowheads). **b** Photomicrograph showing positivity for melanoma-associated antigen 45. **c** Photomicrograph showing positivity for melanin-A

An 82-year-old woman with no previous medical history presented with a black nodule on the vulva. Clinical examination revealed a mass the size of the tip of the index finger on the clitoris that was suggestive of a malignant melanoma (Fig. 1). Abrasive cytology yielded Class IV findings, and an excisional biopsy was therefore performed. Although the resection margins were negative, the tumor was 18 mm thick and had ulcerated, resulting in a classification of pT4b. More extensive resection was considered necessary. A wider margin was created, and sentinel node biopsy was performed (negative). The final diagnosis was malignant melanoma (pT4bN0M0, pStage IIC; UICC 8th edition). Four years later, a routine follow-up computed tomography (CT) scan showed a single 5-mm mass in the lower lobe of the right lung (Fig. 2a). Thoracoscopic partial resection of the lower lobe of the right lung was performed, and pathological examination of the operative specimen confirmed a provisional diagnosis of a pulmonary metastasis of malignant melanoma. The patient was treated with single-agent pembrolizumab as adjuvant chemotherapy for 1 year. A CT scan performed 9 years after the first surgery showed a single 18 mm hypo-absorptive zone in liver segment II (Fig. 2b) with no evidence of lymph node or distant metastasis. T1-weighted magnetic resonance images showed high intensities in the main lesion, suggesting melanin deposition (Fig. 2c). Whole-body F-18-fluoro-2-deoxyglucose (FDG) positron emission tomography–CT revealed mild FDG deposition in the main tumor with a maximum standardized uptake value of 3.76 (Fig. 2d). Taken together, these findings resulted in a provisional diagnosis of a melanoma metastasis. Liver function was good according to the criteria of the Liver Cancer Study Group of Japan, with a Child–Pugh Score of 5 and Liver damage A. Robotic-assisted laparoscopic hepatic left lateral sectionectomy was performed. The operation time was 3 h 43 min, and the blood loss volume was 65 mL. The patient was discharged home with no postoperative complications. Gross examination of the operative specimen revealed a black 2.4- × 2.5 cm tumor in segment II of the liver (Fig. 3). Pathological examination showed tumor cells with melanin pigmentation and oval to spindle-shaped nuclei growing in sheets and nests (Fig. 4a). Immunohistochemistry was positive for melanoma-associated antigen 45 and melanin-A (Fig. 4b, c). The patient was diagnosed with hepatic malignant melanoma metastasis. Pembrolizumab monotherapy was started as postoperative adjuvant chemotherapy for 1 year. At the 1.5 year follow-up, the patient was clinically well with no evidence of recurrence.

## Discussion

We have herein reported a patient who underwent liver resection for a solitary metastasis from a clitoral malignant melanoma, a particularly rare condition. The decision to perform a hepatic resection was based on the likelihood that it would improve the patient’s prognosis.

Cutaneous malignant melanomas account for most malignant melanomas, and mucosal malignant melanomas account for 0.8% to 3.0% of such tumors [5, 6]. Female genital tract melanomas are classified as mucosal melanomas and account for 20% of all mucosal malignant melanomas [7], thus constituting a particularly rare form of female genital tract melanomas. A search of PubMed from 2000 to 2024 for the terms “clitoral malignant melanoma”, “malignant melanoma of clitoris”, and “vulvar melanoma” yielded three reports [8, 9], including our case (Table 1). To the best of our knowledge, this is the first case of treatment of a distant metastasis from a clitoral malignant melanoma.

**Table 1:** Present case and 3 previously reported clitoral malignant melanoma

No	Year	Case report (Reference)	Age	Stage	First surgery	Adjuvant chemotherapy	Follow-up period	Recurrence site	Duration to recurrence	Recurrent therapy
1	2007	Košt’álová, et al.	77	IIC	Local excision of the clitoral mass	–	9 months	–	–	–
2	2019	White, et al.	67	IIB	Local excision of the clitoral mass	–	1 year and 3 months	–	–	–
3	2021	Szlachta-McGinn, et al.	52	IIC	Local excision of the clitoral mass	Pembrolizumab	1.5 months	–	–	–
4	2023	Present case	82	IIC	Local excision of the clitoral mass	Pembrolizumab	9 years	Lung, liver	5 year	Pneumonectomy + AC, Hepatectomy + AC

Malignant melanomas can exhibit simultaneous hematogenous and lymphogenous spread and can metastasize to any organ [10]. Mucosal malignant melanomas are reportedly more likely to give rise to distant metastases than are cutaneous malignant melanomas. The most common sites of distant melanoma metastases are skin/subcutaneous tissue [11], whereas liver, lung, and extra-regional lymph node metastases are more common sites of metastases from mucosal malignant melanomas [12]. The three reported patients who we identified had short follow-up and no recurrence, whereas our patient had recurrences in the lungs and liver, which is consistent with the pattern of recurrence of mucosal melanomas previously reported. Malignant melanoma is known to have the poorest prognosis of all skin cancers. The 5 year survival rate for early-stage (Stage I) malignant melanoma is 91.4%, whereas at 24.6%, the prognosis is relatively poor for patients with distant metastases (Stage IV) [1]. The prognosis of vulvar malignant melanomas is even worse, with 5 year survival rates of 73.6% for Stage I and 3.9% for Stage IV [13]. In this case, there is potential for a favorable prognosis through the resection of oligometastasis and adjuvant chemotherapy.

Molecularly targeted agents or immune checkpoint inhibitors are generally recommended for Stage IV malignant melanoma. However, surgery is also considered for patients with oligometastasis, defined as fewer than five metastases that can be completely resected [2–4]. The recurrence rate of malignant melanoma is as high as 75% and generally requires long-term follow-up for 5–10 years after surgery and short-term follow-up every 3 months for the first 3 years after surgery [14–16]. Despite the high recurrence rate, resection of metastases has been reported to improve overall survival (OS) [2–4]. In a study of resectable Stage IV malignant melanoma, not limited to liver metastases, the surgery group had a significantly greater 4 year survival rate than did the conventional chemotherapy group (20.8% vs. 7.0%, *p* < 0.01) [17]. A meta-analysis of 22 studies involving 579 patients who had undergone resection of malignant melanoma metastases in the liver revealed that OS was significantly longer in the surgery than non-surgery group (14–41 vs. 4–12 months; hazard ratio, 0.32; 95% confidence interval, 0.22–0.46) [2]. Additionally, the interval between resection of the primary tumor and recurrence may be a prognostic indicator. Adam et al. [18] reported that the interval (< 12 vs. > 12 months) between liver metastases and resection of the primary tumor has an impact on 5 year survival in patients with non-colorectal nonendocrine liver metastases (including malignant melanoma) (risk rate, 1.82; confidence interval, 1.47–2.26; *p* = 0.0001). In addition to achieving R0 resection and considering disease-free interval, it is imperative to comprehensively evaluate the sufficient remaining liver volume and the patient’s surgical tolerance to determine the overall surgical candidacy [2]. In recent years, the minimally invasive procedure of robot-assisted surgery has also attracted attention [19–22]. Compared with laparoscopic surgery, robot-assisted surgery is reportedly associated with lower rates of open conversion and shorter postoperative hospital stays [20]. This factor is likely to contribute to the patient’s surgical tolerance as well. Our patient achieved long-term recurrence-free survival (RFS) of 4 years after excision of a lung metastasis. We therefore considered that a favorable outcome would also likely be achieved by resection of a solitary liver metastasis and thus performed robot-assisted excision of the lesion.

In recent years, immune checkpoint inhibitors have been found to be effective when administered as postoperative adjuvant chemotherapy [23, 24]. Consequently, nivolumab and pembrolizumab, human IgG4 monoclonal antibodies against PD-1, were listed for adjuvant chemotherapy of malignant melanoma from 2018 in Japan. To our knowledge, no reports have indicated that such adjuvant chemotherapy is effective specifically for mucosal malignant melanoma; however, pembrolizumab reportedly achieved a significantly longer RFS than ipilimumab and high-dose alpha-interferon (hazard ratio, 0.77; confidence interval, 0.59–0.99; *p* = 0.002), as well as superior safety, with rates of Grade ≥ 3 adverse effects at 19.5%, 49.2%, and 71.2%, respectively [25]. We prescribed pembrolizumab as adjuvant chemotherapy after resection of lung and liver metastases and there has been no recurrence yet. However, clearly superior OS has not yet been reported; future results are awaited.

## Conclusion

We have herein described a patient who underwent resection of an oligometastatic liver tumor from a clitoral malignant melanoma and was treated with postoperative adjuvant chemotherapy. Even patients with liver metastases may achieve prolonged survival if the lesion can be completely resected.

## Data Availability

The data set supporting this article is available upon reasonable request from the corresponding authors.

## Abbreviations

CT: Computed tomography
FDG**-**F: 18-Fluoro-2-deoxyglucose
OS: Overall survival
RFS: Recurrence-free survival
